# Facility Characteristics and Quality Deficiencies of Florida Nursing Homes With COVID-19 Cases and Deaths

**DOI:** 10.1093/geroni/igaa057.3461

**Published:** 2020-12-16

**Authors:** Xiaochuan Wang, Courtney Wilson

**Affiliations:** University of Central Florida, Orlando, Florida, United States

## Abstract

The Coronavirus disease 2019 (COVID-19) has been disproportionately affecting nursing homes throughout the United States, resulting elevated risk for COVID-19 morbidity and mortality to nursing home residents. Given the high percentage of aging population, large number of nursing homes, and staggering surge of COVID-19 cases in Florida, it’s critical to understand factors that may affect Florida nursing homes’ vulnerability to the COVID-19 pandemic. Using Nursing Home COVID-19 Dataset as of July 26, 2020 obtained through Centers for Medicare and Medicaid Services (CMS), and Provider Info Dataset and Health Deficiencies Dataset available through CMS Nursing Home Compare data, we constructed a database of Florida nursing facilities with confirmed COVID-19 cases and deaths, with corresponding facility characteristics and quality deficiencies. We examined the facility characteristics (e.g. facility size, ownership state, chain affiliation, staffing level) and quality deficiencies (e.g. infection control deficiencies) of Florida nursing homes with and without publicly reported COVID-19 cases and deaths. Results indicated that, as of July 26, 2020, 73.3% and 40.8% of Florida nursing homes had resident COVID-19 cases and death, respectively (N=701). Findings also suggested that Florida nursing homes of large facility size, chain affiliated, and for profit, were significantly more likely to have documented resident COVID-19 cases (p<.05). Larger facility size (120 beds or more), staff shortage, and having prior infection control deficiency citation, were significantly related to the odds of having resident COVID-19 deaths (p<.05). Policy and practice implications and future research directions will be addressed to better protect the at-risk nursing home residents.

